# Impedance Pumping and Resonance in a Multi-Vessel System

**DOI:** 10.3390/bioengineering5030063

**Published:** 2018-08-09

**Authors:** Victor Zislin, Moshe Rosenfeld

**Affiliations:** School of Mechanical Engineering, Faculty of Engineering, Tel Aviv University, Tel Aviv 69978, Israel; victorzislin@gmail.com

**Keywords:** impedance pumping, valveless pumping, resonance, multi-vessel system, cardiovascular system

## Abstract

Impedance pumping is a mechanism that generates flow in a compliant vessel by repeatedly actuating the vessel asymmetrically, without employing any internal valves, blades, or other mechanisms. The net flow is obtained by establishing a constructive wave pattern. Elaborate studies of impedance pumping in a single vessel have shown that the flow rate strongly depends on the actuation frequency, as well as on other parameters, such as actuator location and amplitude, and that it operates best in the resonance mode. The present study extends these principles to a network of multiple compliant vessels, representing a cardiovascular system. The flow is modeled numerically by the one-dimensional approximation of the Navier-Stokes equations. Two configurations were examined, systems consisting of three and five compliant vessels. First, the natural frequencies of these configurations were identified. Then, the dependence of the net flow rate (NFR) on the actuating frequency was explored, showing that impedance pumping operates best in the resonance mode in the case of a network of vessels as well. The impact of other parameters were studied as well, such as the location of one or two actuators, actuation amplitude, actuator width, the duty cycle, and the phase lag between the actuators. The results show that impedance pumps can generate significant NFR and the obtained NFR can be manipulated by properly setting up one or more of the governing parameters. These findings indicate that impedance pumping principles may be applied to flow control of the cardiovascular system.

## 1. Introduction

Impedance pumping is a technique to generate, augment, or manipulate flow and/or pressure head in an elastic vessels connected at the edges to vessels with different elastic properties (usually to rigid vessels) [1,2]. Impedance pumps operate by wave reflections caused by impedance mismatch originating from geometrical changes, disturbances, or variations in elastic properties [3,4,5]. Net flow rate (NFR) occurs when the compliant section of the tube is periodically compressed at an asymmetric location at certain frequencies. The operation of impedance pumps requires no bulky moving parts. This design may be a very attractive option in a variety of systems, where flow generation or augmentation is required, and the use of valves or blades is difficult or potentially harmful [3,4,6,7]. It operates in a wide range of sizes down to micro-scale systems [1,3,6,7].

Experimental studies of impedance pumping in closed loop configurations [1,4,7,8] or in open loop configurations [3,7,9] show that for a certain set of parameters, pulsatile flow with significant NFR can be obtained. Moreover, it was appreciated that for certain actuation parameters, the net flow could reverse its direction. Using various actuation characteristics (such as actuator position, frequency, and size), a variety of conditions were presented under which pumping in a flexible tube occurs. In these studies three basic conditions were identified for the net flow to exist in the loop [4]. The first condition was that energy must be provided to the system. The magnitude and frequency of the flow, as well as the efficiency of the whole system strongly depend on the energy waveform [1,5]. The second condition was the presence of a compliant chamber or tube in the loop to allow storage of the displaced fluid. The third condition was that the impedance must alter along the loop’s pathways to generate reflection sites (hence, the name *impedance pump*). Later, it was recognized that nonlinearity is also a vital condition that should be met to generate NFR [1,10].

Unlike peristaltic pumping, where the flow rate is proportional to the pumping frequency, impedance pumps obtain their maximum flow rates close to the natural frequencies of the system indicating that it is most effective when operating in the resonance mode. This was appreciated both experimentally [1,11] and numerically [2,12,13,14,15].

Alongside experimental studies, numerical simulations have become popular in the study of impedance pumping. Full 3D approximation of the fluid-structure-interaction (FSI) problem for a circular cross-section tube have been performed only by Shin and Sung [16], reiterating that NFR depends nonlinearly on the actuating frequency. Simpler 2D approximations are more common [17,18]. The 2D Navier-Stokes equations using the immersed boundary method were employed to solve numerically the flow in closed [8] or open [18] configuration consisting of an elastic section and a rigid section. The flow magnitude and direction were found for a limited number of parameters such as the location, amplitude and frequency of the actuation. Axisymmetric models were studied computationally [5,12] using the finite-element ADINA package, shedding light on the wave-pumping mechanism.

However, 2D, and certainly 3D, FSI simulations of impedance pumping consume large computational resources and, therefore, 1D approximations are common, especially if comprehensive parametric study is pursued [2,8,13,14,19,20,21]. Ottesen [8] employed a 1D approximation of the Navier-Stokes equations to model the flow generated by periodic compression of a torus made of two sections with different stiffness. The FSI phenomena were approximated by an algebraic pressure/area relationship. They conducted experiments as well, which revealed a very good agreement with the numerical results, demonstrating the validity of the 1D approximation. Quite a large volume of studies of impedance pumping employed later the 1-D approximation to study various aspects and, in particular, the dependence of the NFR on the various governing parameters, e.g., [2,13,14,21].

To obtain significant NFR, a large actuation-amplitude is required [2,5,12]. Loumes et al. [12], inspired by the embryonic heart structure, showed that the net flow rate can be augmented in a multi-layer tube of different elastic properties, requiring a smaller actuation amplitude compared to a single layered tube. A different approach to improve the effectiveness of impedance pumping was taken by Rosenfeld and Avrahami [13] employing an array of actuators located at a distances from one another. A numerical simulation solving the 1D version of the Navier-Stokes equations was carried out. They demonstrated that several actuators working close to the natural frequencies and having the appropriate phase delay between them can produce a relatively high net flow rate, even for very small actuation amplitudes. A two-actuator configuration was also studied numerically using the 3D model of Shin and Sung [16]. The dependence of the NFR on the phase between the two actuators, as well as the actuating frequency, were considered. However, the effect of many other parameters that determine the NFR were not studied, probably due to the excessive computational resources required for multi-dimensional FSI simulations.

To date, all the studies exploring impedance pumping employ models of a single compliant tube in an open [13] or closed [2] arrangement. However, the consequences of these studies and possible implementations may go beyond impedance pumping in a single vessel. Impedance pumping techniques can be applied to any flow system consisting of a number of connected vessels of various geometries and elastic properties. In fact, the cardiovascular system is such a case, consisting of a large array of branched vessels of various properties, including various impedance properties. Pahlevan and Gharib [6] have shown, experimentally, for a model of the aorta that impedance pumping may assist in the generation of flow in the cardiovascular system. This study did not consider the cardiovascular system itself but a simplistic model of it.

In the present work the valveless impedance pump concept is extended to a system of interconnected compliant vessels. Several simple configurations of three and five vessels are modeled. The NFR generated at close to resonant mode operation was studied for various governing parameters for the case of one or two actuators placed in various locations. It has been demonstrated that for proper selection of parameters, the net flow rate can be controlled both in magnitude and direction by, for example, varying the actuation frequency or changing the phase-lag between the actuators.

## 2. Materials and Methods

### 2.1. The Physical Model

**Figure 1:**
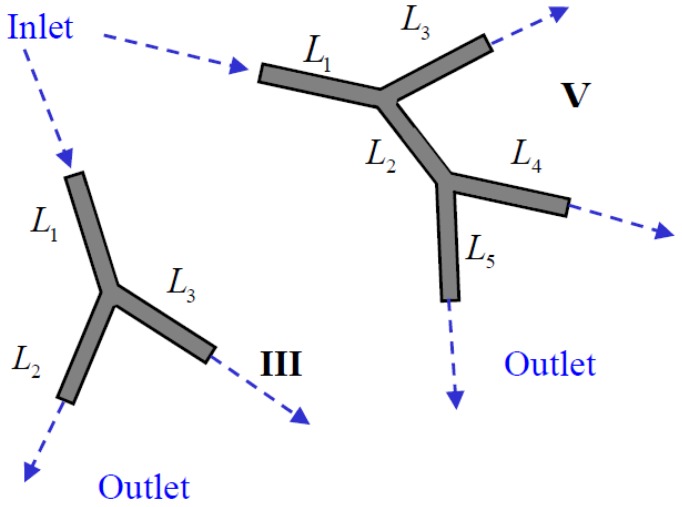
The three-vessels (III) and five-vessels (V) configurations.

The multi-vessel impedance pump consists of a system of interconnected compliant tubes, with one or more actuators. In the present study impedance pumping is considered in a few simple models consisting of three or five vessels (Figure 1) to gain knowledge of the performance of branching vessels systems. The ultimate goal (not of the present article) is to study impedance pumping in the cardiovascular system that consists of a large number of vessels. Yet, it should be emphasized that the vessel configurations studied herein are not intended to model in any way the cardiovascular system.

The fluid is taken to be blood, with density and dynamic viscosity values of $1060\;\mathrm{kg}/m^{3}$ and 0.0035 pa·sec, respectively. To maintain continuity of the velocity at the junctions of the branches, the combined cross-section area of the daughter vessels was chosen to be equal to the cross-section area of the mother vessel, see Table 1 for the vessel lengths and radii. Three main parameters characterize the vessel mechanical properties in the present model: the cross-sectional area of vessel A (or, equivalently, the inner radius of the wall, $R_{0}$), the thickness of the vessel wall h, and the Young’s modulus of the wall, E. To simplify the computational procedures the *elastance* is introduced, similar to Sheng et al. [19]:

(1) $$E_{L}=\frac{Eh}{2R_{0}}$$

where the identical elastance value of $E_{L}=21.5\;\mathrm{Kpa}$ is used in all the vessels considered in the present study.

### 2.2. The Mathematical Model

In the present stage of the study, fundamental concepts and performance issues are being evaluated. To allow a large number of parametric runs of the multi-vessel systems, the flow is modeled by the unsteady 1D, quasi-viscous, incompressible Navier-Stokes equations, which has been shown to yield reasonable results for system of compliant vessels [19,20,21,22,23]. The details of the derivation can be found, for example, in [8,19,22]. This derivation assumes that cross flow and radial variations are negligible and the flow and geometry variations depend only on the axial direction. This relies on the assumption that and the length of the vessels are significantly larger than their hydraulic diameter. Viscosity is modeled by relating the wall shear stress to the mean (axial) flow. The continuity and momentum equations take the form:

(2) $$\frac{\partial A}{\partial t}+\frac{\partial (AU)}{\partial x}=0$$

(3) $$\frac{\partial U}{\partial t}+\frac{\partial }{\partial x}\left(\frac{P}{\rho }+\frac{U^{2}}{2}\right)=F$$

Here, x is the length along a vessel, t is time, A is the cross-sectional area, U is the mean flow velocity at the cross-section A, P is the transmural pressure, and *ρ* is the density. F is the quasi-viscous source term added to the momentum equation to model the friction between the fluid and the vessel wall. For fully developed laminar flow, it can be expressed as:

(4) $$F=-\frac{8\pi \nu U}{A},$$

where *ν* is the kinematic viscosity [8].

The fluid-structure interaction between the flow and the wall of the vessel is modeled by a specified relationship between the pressure and the cross-sectional area, P=P(A), the so called *state equation*. Based on the linear theory of elasticity, the following relation between the transmural pressure and the cross-sectional area is selected [8,22]:

(5) $$P-P_{0}=\frac{8}{3}E_{L}\left(1-\sqrt{\frac{A_{0}}{A}}\right)$$

where $P_{0}$ is a reference pressure and $A_{0}$ is the cross-sectional area at rest (when $P=P_{0}$). Other state equations have been suggested, such as in [19], but many studies in impedance pumping adhere to Equation (5) [2,8,21,22].

In the present preliminary study, at the entrance to the system, as well as at the outlets, a constant (zero) pressure boundary condition is applied (that also means that the vessels are rigidly connected at these points). Our interest is to explore the performance of “pure” impedance pumping without any enforced pressure drop. For specified pressure boundary condition, the traveling pressure waves are fully reflected back into the system. Since the operation of impedance pumping heavily depends on the wave dynamics it is vital to model the wave reflections at the boundaries and at the junction of the branches accurately. Therefore, following several previous studies [14,19,20], the method of characteristics is adopted for applying the boundary conditions and the conditions at the junctions of the branches. Given the pressure, the velocity at the boundaries is determined from the characteristic equations. At the branches’ junctions, flow and pressure continuity are enforced between the mother and the daughter vessels [19].

### 2.3. The Actuation Mechanism Model

The actuating mechanism of the compliant vessel inserts power into the system by generating pressure waves along the vessel. In the present 1-D approximation, it is modeled by imposing periodic variation to the undisturbed cross-section area of the vessel $A_{0}$ at the location of the actuator [8,13]. It is equivalent, see Equation (5), to specifying the actuation pressure. For a system with multiple actuators, it takes the form:

(6) $$A_{0}{}_{a}(x,t)=A_{0}{}_{a}\left(1+X_{a}(x)\cdot T_{a}(t)\right)$$

where a is the index of the actuator and $A_{0}{}_{a}$ is the specified undisturbed cross-section area of the vessel on which the actuator is installed. $X_{a}(x)$ and $T_{a}(t)$ are defined by the following sine functions:

(7) $$X_{a}(x)=\left\{ \begin{array}{cc} -\alpha_{a}\cdot \sin\left(\frac{\pi }{w_{a}}(x-x_{0a})\right) & x_{0a}\leq x\leq x_{0a}+w_{a}\\ 0 & else\; \end{array}\right.$$

and:

(8) $$T_{a}(t)=\left\{ \begin{array}{cc} \sin\left(\frac{f_{a}}{DC_{a}}\pi (t-t_{0a})\right) & t_{0a}\leq t\leq t_{0a}+\frac{DC_{a}}{f_{a}}\\ 0 & else \end{array}\right.$$

**Figure 2:**
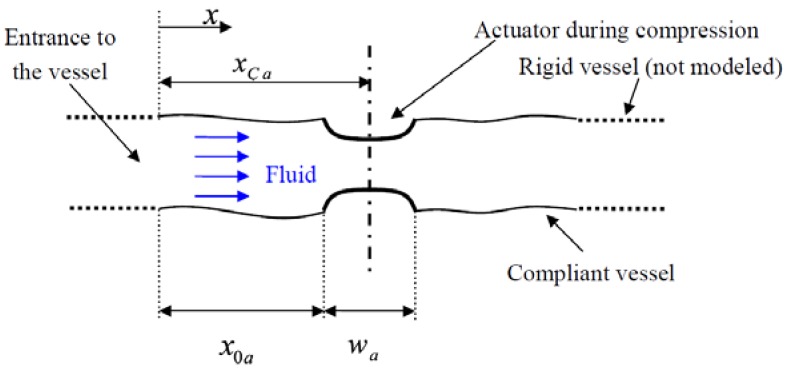
A sketch of a compliant vessel with an actuator and the notations used.

The parameters of the actuator a are the relative actuation amplitude $\alpha_{a}$, the width of the actuator $w_{a}$, the upstream coordinate of the actuator $x_{0a}$, the actuation frequency $f_{a}$, the actuator duty cycle $DC_{a}$ and the time delay from the previous actuator $t_{0}{}_{a}$, see Figure 2 for a sketch of a vessel with an actuator. The actuator duty cycle, $DC_{a}$ is the relative duration of the actuator’s operation within one time cycle. The phase delay of the actuator is given by $\pi t_{0a}f_{a}/DC_{a}$.

Unless otherwise mentioned, an actuator width of $w_{a}=1\;\mathrm{cm}$ and a relative actuation amplitude of $\alpha_{a}=0.1$ are employed for all the actuators using a duty cycle of $DC_{a}=1$. The locations of the actuators are explicitly given for each case.

### 2.4. Computational Methods

**Figure 3:**
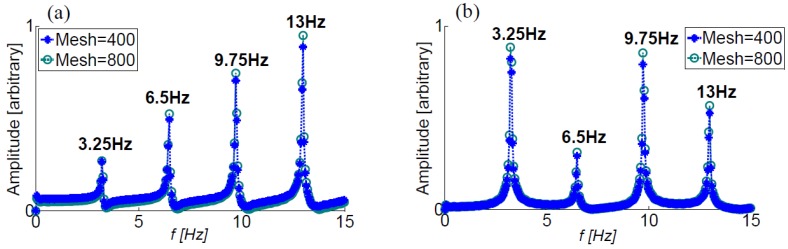
The FFT of the pressure signals at the entrance to the first (**a**) and the second (**b**) vessels calculated for the three equal-length vessels configuration (III-a, Table 1). Mesh refinement study results are also shown.

The hyperbolic 1D fluid motion Equations (2) and (3) are solved numerically using the Lax-Wendroff method, which is a two-step explicit scheme of second order accuracy both in space and time. This method was chosen due to its eminent success in previous studies in the field [13,19,22]. The computations have initialized from a zero-velocity initial condition and were carried out until a repetitive time-periodic motion was obtained. At each time step, the variables at the vessels boundaries (entrance, exit and branch junctions) were first updated, followed by the update of the inner field points. A small value of numerical diffusion was added to smooth out the results. For the spatial discretization, a mesh size of Δx=1 mm and a time step of $\Delta t=10^{-4}\;s$ were used, resulting in a typical Courant–Friedrichs–Lewy number of 0.6. These values were determined following mesh and time-independence tests (see Figure 3 for the results of a mesh-independence test).

The calculations were performed using a self-written code in C using Microsoft Visual C++ 2008 Express Edition freeware (Microsoft, Redmond, WA, USA). All data acquisition and imaging were performed using Matlab (MathWorks, Natick, MA, USA).

## 3. Results

The interest of the present study is in the net flow rate (NFR) generated by the multi-vessel impedance pump operating at zero pressure drop. The dependence of the NFR on several parameters is elaborated in the following sections.

### 3.1. Natural Frequencies of a Multi-Vessel Compliant System

For a single-vessel, it was shown that the behavior of an impedance pump is strongly dependent on the natural frequency of the vessel [1,12,13]. Significant NFR is obtained only when the pump is operating in resonance mode. Therefore, the natural frequencies of the systems were identified numerically. Each configuration was impulsively actuated by a pressure impulse at the entrance to the system (10^−3^ s long with a magnitude of 6600 pa), and the pressure at certain key points was monitored in time. A Fast Fourier Transform was performed to identify the natural frequencies of the system.

For symmetric boundary conditions (given pressure at inflow and outflow as used in the present study) the theoretical fundamental natural frequency of a single compliant vessel can be approximated by the rate at which a weak wave disturbance would travel back and forth the length of the vessel [14]:

(9) $$f_{1}=\frac{c_{0}}{2L}$$

where $c_{0}$ is the linear wave speed and L is the vessel length. The wave speed is calculated from $c^{2}={\frac{A}{\rho }\frac{dP}{dA}|}_{A=A_{0}}$ [19]. Using Equation (5), the wave speed is given by $c_{0}=\sqrt{\frac{4}{3}\frac{E_{L}}{\rho }}$. In the present model $c_{0}=5.2\frac{m}{s}$ was chosen in all the cases, typical to the wave speed of large arteries in the cardiovascular system.

A single wave traveling along a vessel in a multi-vessel system will encounter a branch junction or a peripheral boundary. The wave will partially (or fully) reflect and travel back into the vessel, while the remaining wave is transmitted downstream to the following vessels in case of a branch, or out of the system in case of an outlet boundary. A *path* is defined as a combination of vessels along which a pressure wave may travel back and forth. For example, in configuration V (Figure 1), a few possible paths can be found: the combination of vessels 1 and 3 or vessels 1, 2, and 5, or vessel 4 alone, etc.

The arguments leading to the estimation of the fundamental natural frequency of a single vessel as elaborated in [14] are extended in the present study to a branched system of vessels. It is assumed that one needs to account for all the possible paths present in the system to estimate all the fundamental frequencies using Equation (9), resulting in several fundamental natural frequencies for each configuration. As will be shown below, this assumption yields a good agreement between these theoretical predictions to the calculated values.

Configuration III-a, consisting of three equal-length vessels, is considered first (see Figure 1 and Table 1). A wave can travel back and forth along two possible paths: a single vessel (either one of the three identical vessels) with a path length of 0.4 m × 2; or a combination of two vessels (vessels 1 and 2 or vessels 1 and 3), with the path of (0.4 m+0.4 m)×2. The theoretical natural frequencies using (9) for each path length yields t 3.25 and 6.5 Hz and their multiples and sums. The calculated natural frequencies in Figure 3 agree well with the theoretical values, proving the applicability of the present approach for in the case of multiple branched frequencies

Results of a finer mesh are also shown with negligible effect. Similar grid-dependence tests were conducted for other configurations as well, but are not presented herein.

**Figure 4:**
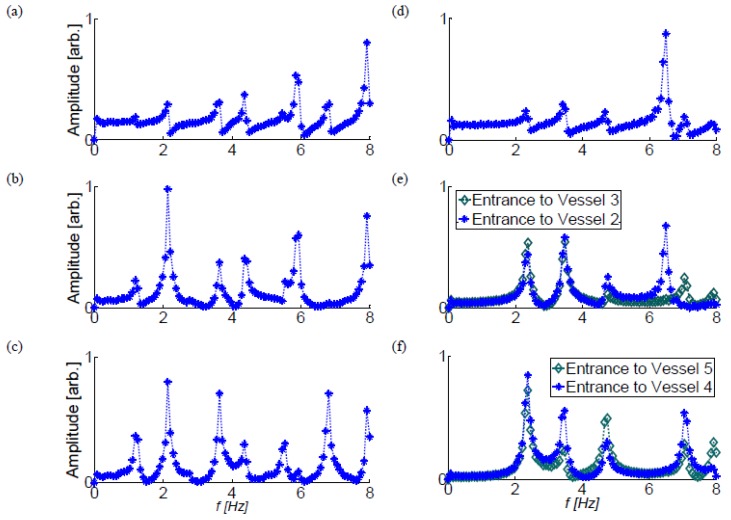
The FFT of the monitored pressure at the entrance to the various vessels of configurations III-b and V. (**a**–**c**) represent the first, the second and the third vessels in configuration III-b, respectively, (**d**) shows the first vessel in configuration V, and (**e**,**f**) the other four vessels in configuration V.

In configurations III-b and V the variability of the possible paths along which pressure waves can travel back and forth is larger (Table 2) and the estimated theoretical frequencies are closer to each other. Additionally, at the branch junctions, only partial wave reflections occur, so that it is more difficult to predict the natural frequency of a system with a larger number of branches. Nevertheless, for configurations III-b and V, a good agreement is still obtained between the calculated (Figure 4) and the theoretical values (Table 3).

### 3.2. Impedance Pumping in Multi-Vessels Systems

Once the natural frequencies of the various configurations are identified, the dependence of the NFR on a selected range of parameters is studied for the case of a zero imposed pressure drop. Unlike in peristaltic pumps where the flow rate is proportional to the pumping frequency, impedance pumps obtain their maximum flow rates close to the natural frequencies of the system [1,2,5,13,14]. Therefore, the effect of the actuation frequency on the NFR is examined first, mainly to verify that impedance pumping performs best in the resonance mode in the case of multiple vessels as well. Next, the dependence of the NFR on other parameters, such as the location of the actuators, the duty cycle, the actuator amplitude, and width is presented. In the case of a system with two-actuators, the location and the phase lag between the actuators is studied. The goal of the parametric studies is to demonstrate how these parameters affect the NFR. A full parametric study is beyond the scope of the present study. The NFR is shown dimensionally as in other similar studies [8,12,13) since a physically-reasonable quantity to scale with is absent.

#### 3.2.1. Three-Vessels Model

**Figure 5:**
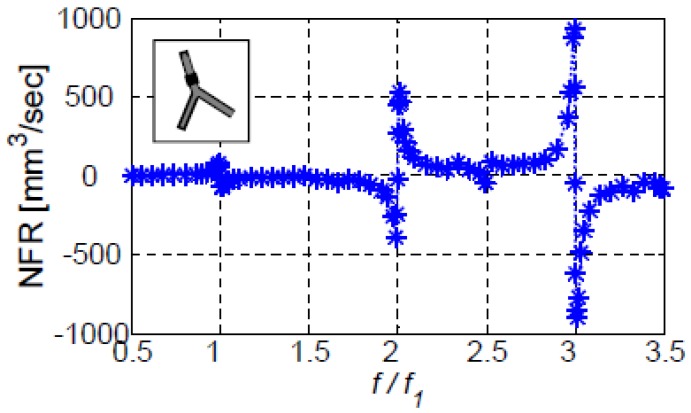
The dependence of NFR on the normalized actuation frequency for configuration III-a. The frequency is normalized by the first fundamental frequency of III-a, f_1_ = 3.25 Hz. The actuator was located on the first vessel at $X_{C}/L_{1}=0.75$.

**Figure 6:**
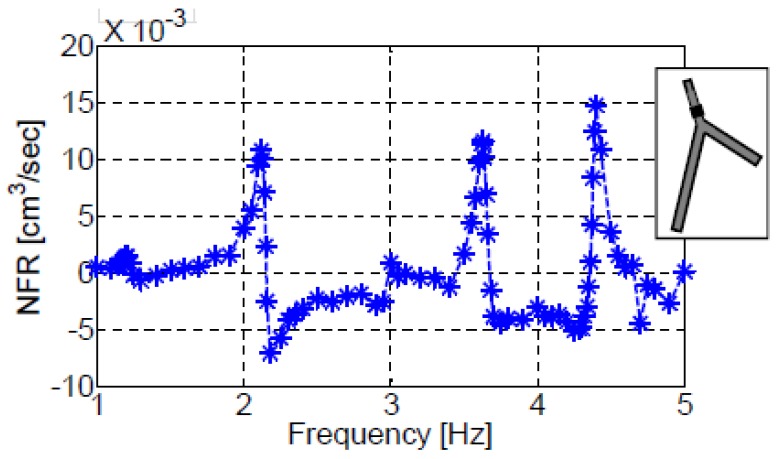
The dependence of NFR on the actuation frequency for configuration III-b. The actuator was located on the first vessel at $X_{C}/L=0.75$.

For an actuator located on the first vessel ($X_{C}/L_{1}=0.75$), Figure 5 and Figure 6 present the dependence of the NFR on the actuation frequency for configurations III-a and III-b, respectively. In these figures and the following ones, the frequency is normalized by the first natural frequency of each configuration. Significant NFR is obtained only in the vicinity of the natural frequencies of each system: $f/f_{1}=1$, $f/f_{1}=2$, etc., for configuration III-a and $f/f_{1}=1$, $f/f_{1}=1.8$, $f/f_{1}=2.33$, and $f/f_{1}=3.05$, etc., for configuration III-b. At other frequencies negligible values of NFR are observed. This is in agreement with previous findings for the case of a single vessel. The NFR values obtained for configuration III-a are more than an order of magnitude larger than those of configuration III-b. This is related to the longer vessels of configuration III-b (Table 1). Longer vessels reduce the NFR (for the same actuation) due to several factors. First, longer vessels suffer from larger viscous losses that result in less kinetic energy and lower NFR. Second, the longer vessels store more elastic energy in the walls and consequently less energy is delivered to the fluid. Third, the residual energy needs to drive a larger mass of fluid reducing velocity and, therefore, NFR.

**Figure 7:**
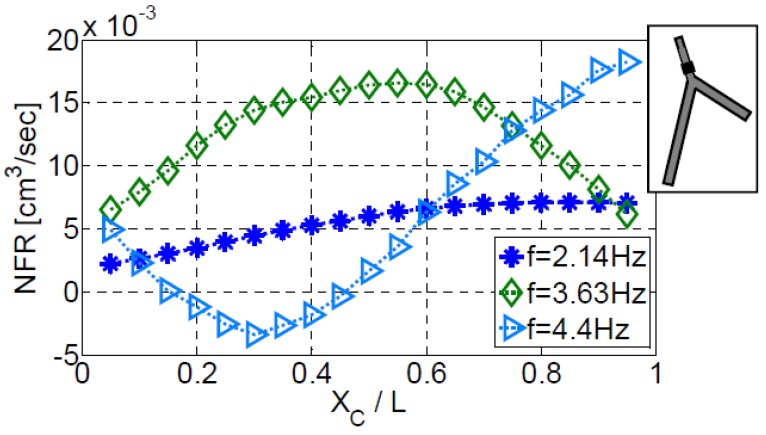
The dependence of NFR on the actuator location positioned on the first vessel of configuration III-b. Three actuation frequencies were examined.

Figure 7 shows the variation of the NFR with the location of the actuator on the first vessel of configuration III-b. Three actuation frequencies are shown with the values of $f/f_{1}=1.8$, $f/f_{1}=3.05$, and $f/f_{1}=3.7$, close to the second, third, and fourth natural frequencies, respectively. The shape of the NFR curve is different for each of the three frequencies, and the maximum NFR is obtained at different actuator locations. Moreover, for the actuation frequency of $f/f_{1}=3.7$, negative flow is observed when positioning the actuator at $0.15\leq X_{C}/L_{1}\leq 0.45$. For the other two frequencies, no negative flow is established. These findings clearly indicate the non-linear behavior of the system.

The duty cycle has been found to have a significant effect on the NFR [1,11,15]. Periodic actuation of an elastic vessel should add power to the fluid. The power added to the fluid is given by:

(10) $$Power=-\int_{x_{0a}}^{x_{0a}+w_{a}}\left(P-P_{0}\right)\frac{dA}{dt}dx$$

**Figure 8:**
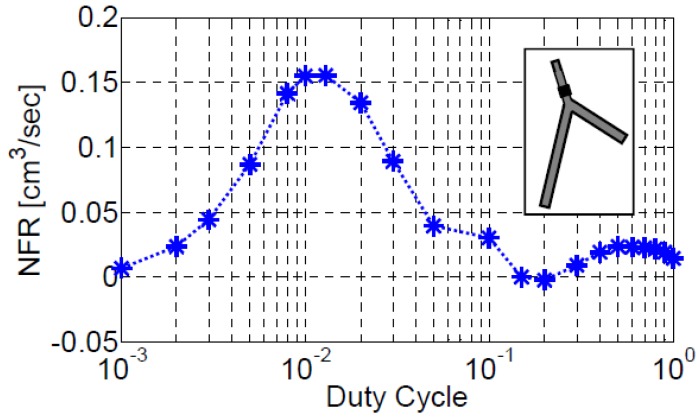
The dependence of NFR on the actuator duty cycle for configuration III-b. The actuator was located on the first vessel at $X_{C}/L=0.75$. Actuation frequency was 4.4 Hz, close to the fourth natural frequency of the system.

Thus, positive power is added to the fluid in the actuation region if the cross-section area decreases when the fluid pressure is greater than the reference pressure (or if the cross-section area increases but the pressure is less than the reference pressure). Otherwise, power is extracted from the fluid by the actuator. Thus, to invest more power into the system (and increase NFR) the actuation duty cycle should be synchronized with the generated wave pattern that determines the cross-section area. Consequently, the duty-cycle that maximizes the NFR is case dependent. Figure 8 reveals that fixing the duty cycle to the optimal value can increase the NFR relative to the case with a duty cycle of 1 by more than one order of magnitude, i.e., from a value of 14.5 mm^3^/s to a value of 155.2 mm^3^/s at duty cycle of $10^{-2}$. Nevertheless, in most of the present simulation a duty cycle of 1 was employed (at the price of obtaining low NFR) since our interest is in finding trends, not in maximizing the NFR.

**Figure 9:**
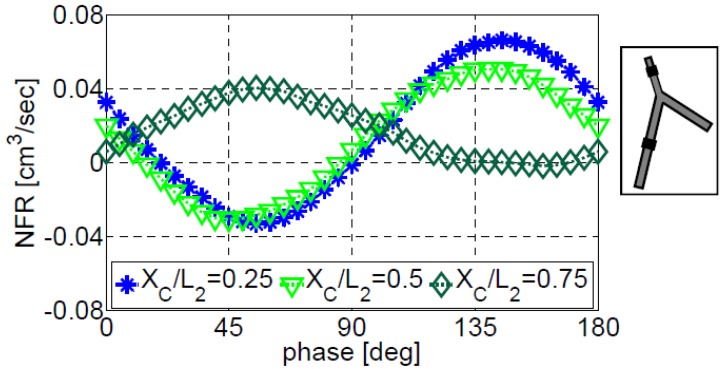
The dependence of the NFR on the phase lag between two actuators located on vessels #1 and #2 of configuration III-b. The first actuator was located on vessel #1 at $X_{C}/L_{1}=0.75$. Three locations of the second actuator are presented. The actuation frequency was 4.4 Hz, close to the fourth natural frequency of the system.

Multiple actuators generate multiple waves that travel in the system, partially reflecting at the branch junctions and at the peripheral boundaries and, also, interacting between themselves. By changing the phase between the actuators, constructive wave pattern can be potentially generated, increasing NFR by proper synchronization (phase) between the actuators. Figure 9 shows the impact of the phase between two actuators on the NFR of configuration III-b. The first actuator is placed on vessel #1 at $X_{C}/L_{1}=0.75$, while the second actuator is placed on vessel #2 at three different locations, $X_{C}/L_{2}=0.25$, 0.5, and 0.75. The frequency of all actuators is $f/f_{1}=3.7$. The dependence of the NFR on the phase is similar to that obtained for two actuators on a single vessel [13]. The plots in Figure 9 are not symmetrical, as the actuators are not located symmetrically. The location of the second actuator has a pronounced effect. The NFR can be increased significantly and even reverse its direction by only changing the phase between the actuators. For example, for the second actuator located at $X_{C}/L_{2}=0.75$ operating at a phase of 55° relative to the first actuator, the NFR is increased almost ten-fold from the value at zero phase, implying the strong effect of the phase between the actuators on the NFR. Moreover, it can be seen that the NFR magnitude and its direction can be controlled solely by changing the phase- between the actuators. The use of the second actuator increases the NFR by a factor of 5 relative to a single actuator on vessel #1 (from 13 mm^3^/s to 65 mm^3^/s).

#### 3.2.2. Five-Vessels Model

**Figure 10:**
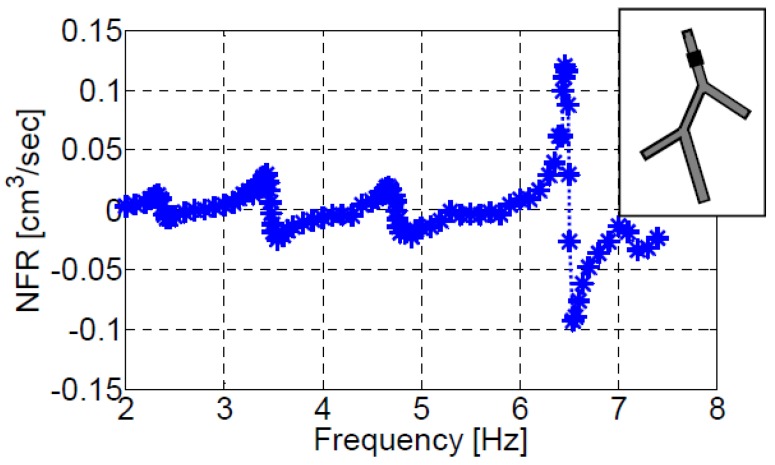
The dependence of NFR on the actuation frequency for an actuator positioned on the first vessel of configuration V, at $X_{C}/L=0.4$.

**Figure 11:**
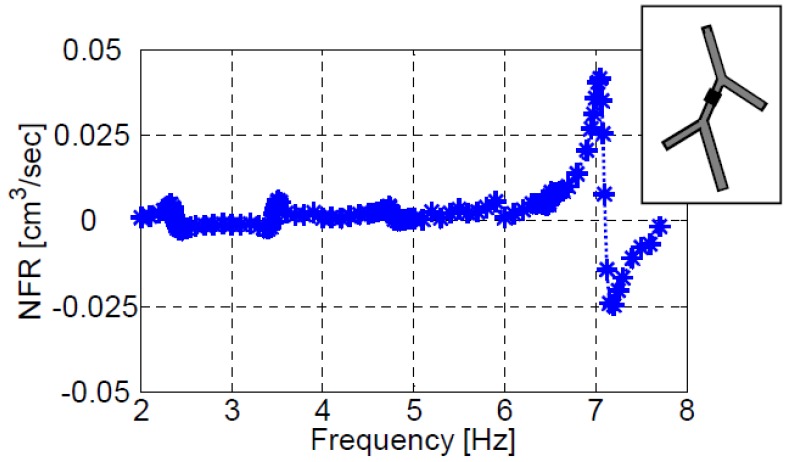
The dependence of NFR on the actuation frequency for an actuator positioned on the second vessel of configuration V ($X_{C}/L=0.2$).

Similar to the three vessels case, the five vessels model provides the larger NFR at the resonance mode, Figure 10. The natural frequencies of the five vessels system clearly stand out, and the maximum positive (or negative) NFR are obtained at their vicinity in the case of an actuator located on the first vessel ($X_{C}/L_{1}=0.4$). Figure 11 shows the impact of the actuation frequency on the NFR for an actuator positioned on the second vessel ($X_{C}/L_{2}=0.2$). In this case only the fifth natural frequency results in a notable NFR. Positioning the actuator at other locations, does not affect the resonant behavior of the system, but only the magnitude of the NFR. The lower NFR obtained relative to three-vessel configurations is a consequence of the overall larger length (and volume) of the five-vessel configuration, as elaborated above for configuration III-b.

**Figure 12:**
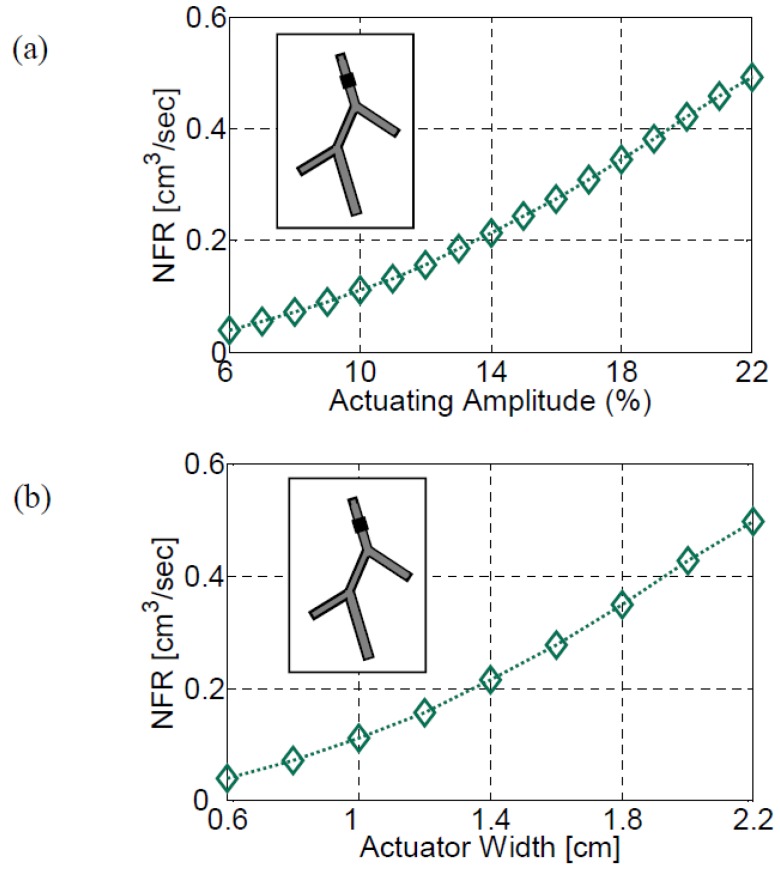
The effect of the actuation amplitude (**a**) and the actuator width (**b**) for configuration V. The actuation frequency was f = 6.42 Hz. For the study of amplitude effect on the NFR (**a**), the width of the actuator is $W_{p}=1\;\mathrm{cm}$. For the study of actuator width effect (**b**) the actuation amplitude is 10%. The actuator was located at $X_{C}/L=0.4$ of the first vessel.

The variation of the NFR with the actuator amplitude ($\alpha_{a}$) and width ($w_{a}/L_{1}$) is presented in Figure 12a,b, respectively, for an actuator located on vessel #1 at $X_{C}/L_{1}=0.4$. The actuation frequency was close to the fourth natural frequency of this configuration. It has been previously shown by Rosenfeld and Avrahami [13] that an increase in either the amplitude or the actuator width results in an increase in the NFR. Similarly, the NFR in the present case increases monotonically with either the amplitude or the width of the actuator in the case of five vessels system, as well, since both of these parameters have a similar impact on the generated pressure waves.

**Figure 13:**
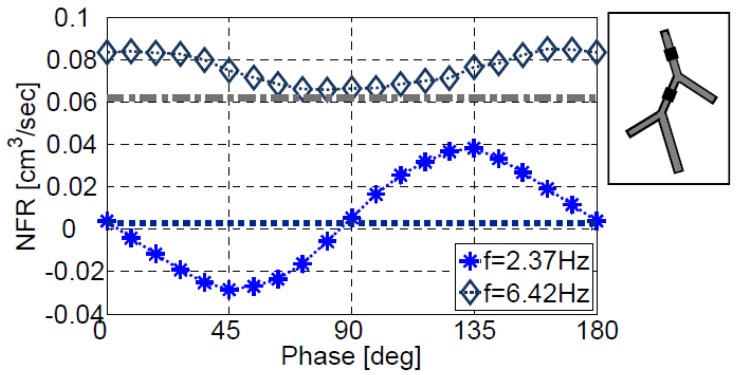
The effect on the NFR of the phase between two actuators positioned on the first and the second vessels of configuration V. The first actuator is located at $X_{C}/L_{1}=0.4$ of the first vessel and the second actuator is positioned at $X_{C}/L_{2}=0.8$ of the second vessel. The dotted and the dash-dotted lines show the NFR for a single actuator positioned at $X_{C}/L=0.4$ of the first vessel.

Figure 13 presents the effect of the phase between two actuators positioned on the first and the second vessels of configuration V at $X_{C}/L_{1}=0.4$ and $X_{C}/L_{2}=0.8$, respectively. Two operating frequencies are compared, $f/f_{1}=1$ (Figure 13) and $f/f_{1}=2.71$ (Figure 13). For $f/f_{1}=1$ the phase has a significant influence on the NFR. The NFR reaches a maximum value of 38.1 mm^3^/s for a phase delay of 135°, showing an increase of more than an order of magnitude compared to the zero phase delay NFR value of 3.76 mm^3^/s or 2.65 mm^3^/s obtained for a single actuator positioned on the first vessel at the same location. Thus, by proper synchronization, the operation of two actuators may results in a significant increase in the NFR, beyond the sum of the two individual actuators.

The NFR shows an anti-symmetric dependence on the phase around 90° and negative flow rates are obtained for most of the range from 0° to 90°. For $f/f_{1}=2.71$ (Figure 13) higher NFR values are obtained with a symmetric dependence on the phase around 90°. The dependence of the NFR in the phase is moderate, with values ranging from 1.05 to 1.37 times the NFR value of a single actuator located at the same location and operating at the same frequency, with a more limited ability to control the NFR relative to the previous case of $f/f_{1}=1$. A proper combination of the actuation frequency and phase lag between two actuators can be useful in a number of applications, as the NFR can be easily controlled by varying either one of the two parameters.

**Figure 14:**
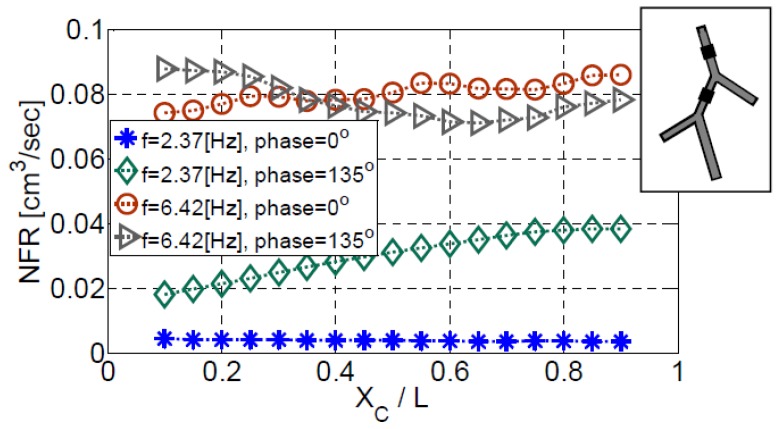
The dependence of the NFR on the position of the second actuator for configuration V with two actuators installed on vessel #1 and #2 while the first actuator is located at $X_{C}/L_{1}=0.4$ Two actuation frequencies are presented, 2.37 Hz and 6.42 Hz. Results are shown for simultaneously-operating actuators, as well as for a phase value of 135° (optimal value for f = 2.37 Hz, Figure 13).

Figure 14 presents the variation of the NFR with the position of an actuator located on vessel #2, while another actuator on vessel #1 is fixed at $X_{C}/L_{1}=0.4$. Two frequencies ($f/f_{1}=1$, Figure 14 and $f/f_{1}=2.71$, Figure 14) are shown. Operating at the first natural frequency and zero phase (Figure 14), the actuator position on vessel #1 has a minor effect on the NFR. The mean NFR value is 3.9 mm^3^/s while the NFR value obtained with a single actuator on vessel #1 positioned at the same location and operating at the same frequency is 2.65 mm^3^/s, indicating that the first actuator dominates the flow formation in this case. By employing a phase of 135°, the NFR can be increased by an order of magnitude with a maximum value of 38 mm^3^/s, positioning the second actuator at $X_{C}/L_{2}=0.9$. By pumping the system at the frequency of $f/f_{1}=2.71$ (Figure 14) a further improvement is obtained. Without a phase between the actuators, a maximum value of 86.2 mm^3^/s is obtained positioning the actuator at $X_{C}/L_{2}=0.9$ while for a phase value of 135° a maximum NFR of 88 mm^3^/s can be obtained. As was already mentioned, the phase has a moderate impact on the NFR for a higher actuation frequency of $f/f_{1}=2.71$, Figure 13. Therefore, for any given position of the second actuator, there is only a small difference between the NFR values obtained for phases of 0° and 135°.

## 4. Discussion

In recent years, impedance pumping has gained popularity and was extensively studied for the case of a single vessel [1,3,6,7,8,9,14,15,18,21]. However, impedance pumping in a system of vessels has not yet been studied. Considering possible bio-medical implications, impedance pumping in a system of multiple compliant vessels connected in parallel and in series is of great interest. As an initial step, some aspects of impedance pumping in a few multi-vessel systems consisting of three or five elastic vessels were examined in the present work.

The simulations employed a simplified 1-D approximation of the Navier-Stokes equations to allow the execution of a large number of simulations for exploring the effect of various parameters on the net flow rate (NFR) in the case of systems consisting of multiple vessels. Based on previous experience of such models for a single compliant vessel [8,19,22], it can be assumed that the main features of the flow phenomena are reasonably well captured. In particular this assumption is valid for estimating global quantities, such as the NFR. A multi-dimensional model might improve accuracy, model better viscous effects and capture secondary phenomena, but it might prohibit extensive parametric studies due to the very large computer resources required for simulating fluid-structure interaction models in multi-vessel systems. Indeed, existing 2-D and 3-D studies of impedance pumping consider only very few cases without an extensive parametric study even for the single vessel cases [5,12,16,17,18].

The frequency response analysis proves that a linear estimation of the natural frequency proposed in [14] for a single compliant vessel can be extended to predict the natural frequencies of a multi-vessel system as well. Their model states that the natural fundamental frequency is $f=c_{0}/2L$ for symmetric (same type) boundary conditions ($c_{0}$ is the linear wave velocity and L is the vessel length). This simplified model yielded in the present study good predictions of the natural frequencies of the multi-vessels systems as well, provided that L represents all possible waves’ paths along the various combination of vessels. This extension is a novelty of the present study.

Analogously to the published results investigating impedance pumping in a single compliant vessel [5,10,13], the present results show that significant NFRs are obtained only at pumping frequencies that are close to the natural frequencies of the system, while at other frequencies the net flow rate is low or even negligible. This feature was observed in all multi-vessel systems examined in the present study as well. Therefore, to yield significant NFR, impedance pumps should operate in the case of multi-vessel systems in resonance mode as well. This is yet another novel finding of this work.

Modifying the location of an actuator operating near to one of the natural frequencies, has a pronounced effect on the NFR. The proper location of an actuator for maximizing NFR, depends on the vessel position in the system that the actuator is operating. The combination of the actuation frequency and optimized location of the actuator can improve significantly the NFR of the system, Figure 7 and Figure 14. The actuation amplitude and the width of the actuator exhibit a very similar behavior on the characteristics of the pump for the case of a network of compliant vessels as well. Increasing the value of either the amplitude or the actuator width will results in an increase of the NFR. Another effective way for manipulating the magnitude of the NFR is controlling the duty cycle, Figure 8.

The use of more than one actuator was found to be very useful for augmenting, as well as for controlling, the magnitude and direction of the NFR in the case of a single vessel [5,13]. The present study reaches to similar conclusions for a two-actuator arrangement, each actuator on a different vessel.

## 5. Conclusions

The present study explored, for the first time, impedance pumping in a multi-vessel system employing a 1-D approximation of the fluid structure interaction problem. Similar to the single vessel case, it was confirmed that significant NFR (net flow rate) can be obtained only when the pumping frequency is near one of the natural frequencies of the system, i.e., in resonance mode. It was also found that in the multi-vessel cases studied, the actuation location, duty cycle, and amplitude, the width of the actuator, as well as the number of actuators and the phase delay between them have a substantial effect on the NFR, similar to previous finding for a single vessel system.

To obtain significant NFR, the amplitude and actuators’ size should be increased and the actuators should be located properly (depending on the vessel branch as well) and the duty cycle selected carefully. The number of actuators and the phase between them significantly affect the NFR magnitude and direction as well.

The limited parametric study of this work has shown that impedance pumping concepts can be extended to the case of multiple vessels connected in parallel and in series. The actuation frequency, the phase delay, and the duty cycle are much more manageable than the other attributes of impedance pumping (such as the number, the width, or the location of the actuators). Properly selected parameters can augment the flow rate significantly in many situations.

In fact, the phase delay between the actuators can be utilized very conveniently to control the NFR magnitude, as well as its direction. This property can be very beneficial in cases where real-time regulation of the net flow is required. For example, a failing heart with insufficient blood flow might be assisted with two or more actuators which regulate the blood flow in the circulation by changing the phase between the actuators. Another obvious benefit of impedance pumping is the ability to place the actuators on the outer part of the vessel, facilitating their installation with minimal damage to the wall of the vessels or to the fluid flowing inside the vessels (such as blood).

## Figures and Tables

**Table 1:** The lengths and the radii of the three and five-vessel configurations.

	Length	Radius
Configuration III-a Identical Lengths	$L_{1}=40\;\mathrm{cm}$ $L_{2}=40\;\mathrm{cm}$ $L_{3}=40\;\mathrm{cm}$	$r_{1}=1\;\mathrm{cm}$ $r_{2}=0.71\;\mathrm{cm}$ $r_{3}=0.71\;\mathrm{cm}$
Configuration III-b Different Lengths	$L_{1}=40\;\mathrm{cm}$ $L_{2}=90\;\mathrm{cm}$ $L_{3}=190\;\mathrm{cm}$	$r_{1}=1\;\mathrm{cm}$ $r_{2}=0.71\;\mathrm{cm}$ $r_{3}=0.71\;\mathrm{cm}$
Configuration V	$L_{1}=40\;\mathrm{cm}$ $L_{2}=40\;\mathrm{cm}$ $L_{3}=40\;\mathrm{cm}$ $L_{4}=70\;\mathrm{cm}$ $L_{5}=30\;\mathrm{cm}$	$r_{1}=1\;\mathrm{cm}$ $r_{2}=0.71\;\mathrm{cm}$ $r_{3}=0.71\;\mathrm{cm}$ $r_{4}=0.5\;\mathrm{cm}$ $r_{5}=0.5\;\mathrm{cm}$

**Table 2:** A list of the possible paths and the theoretical natural frequencies.

**Configuration III-b**		
Vessels	Path Length (m)	Theoretical Frequency (Hz)
1	0.4	6.5
2	0.9	2.89
1 and 2	1.3	2
3	1.9	1.37
1 and 3	2.3	1.13
**Configuration V**		
Vessels	Path Length (m)	Theoretical Frequency (Hz)
5	0.3	8.67
1 or 2 or 3	0.4	6.5
4 or 2 and 5	0.7	3.7
1 and 2 or 1 and 3	0.8	3.25
1 and 2 and 5 or 2 and 4	1.1	2.36
1 and 2 and 4	1.5	1.73

**Table 3:** Comparison of the calculated and theoretical natural frequencies.

**Configuration III-b**	
Calculated Frequency (Hz)	Theoretical Frequency (Hz)
1.19	1.13 1.37
2.14	2 2.26 (1.13 × 2)
2.77	2.89 2.74 (1.37 × 2)
3.64	3.39 (1.13 × 3) 4 (2 × 2)
4.36	4 (2 × 2) 4.11 (1.37 × 3) 4.52 (1.13 × 4)
5.55	5.48 (1.37 × 4) 5.65 (1.13 × 5)
5.9	5.78 (2.89 × 2) 6 (2 × 3)
6.8	6.5 (6.5 × 1) 6.85 (1.37 × 5)
**Configuration V**	
Calculated Frequency (Hz)	Theoretical Frequency (Hz)
2.37	2.36
3.49	3.25 3.7
4.75	4.72 (2.36 × 2)
6.5	6.5 6.5 (3.25 × 2)
7.05	7.08 (2.36 × 3)
7.92	7.4 (3.7 × 2) 7.97 (4.72 + 3.25) 8.67
